# Behavioral and Developmental Responses of *Habrobracon hebetor* (Hymenoptera: Braconidae) to Larvae of *Helicoverpa armigera* (Lepidoptera: Noctuidae) Inoculated With Various Concentrations of *Bacillus thuringiensis* var. *kurstaki* (Bacillales: Bacillacae)

**DOI:** 10.1093/jisesa/ieaa129

**Published:** 2020-11-24

**Authors:** Rahim Allahyari, Shahram Aramideh, J P Michaud, Mohammad Hassan Safaralizadeh, Mohammad Reza Rezapanah

**Affiliations:** 1 Department of Plant Protection, Urmia University, Urmia, Iran; 2 Department of Entomology, Kansas State University, Agricultural Research Center-Hays, Hays, KS; 3 Department of Biological Control, Iranian Research Institute of Plant Protection, Agricultural Research Education and Extension Organization, Tehran, Iran

**Keywords:** biological control, entomopathogen, host selection, parasitism, survival

## Abstract

*Bacillus thuringiensis* Berliner subsp*. kurstaki* (*Btk*) and *Habrobracon hebetor* Say are both biological control agents of *Helicoverpa armigera* Hubner. The present study evaluated their compatibility for combined application against this pest by examining the acceptability of *Btk*-inoculated hosts for *H. hebetor* females and testing for negative life-history impacts on developing progeny. Second-instar *H. armigera* larvae fed for 72 h on potted chickpea plants treated with three concentrations of *Btk* (LC_15_, LC_35_, and LC_70_) and were then used in bioassays of parasitoid development and parasitism behavior. Survival of parasitoids was significantly reduced, and immature development prolonged, on hosts fed chickpea plants treated with LC_35_ and LC_70_  *Btk*, but not on plants treated with LC_15_  *Btk*. Parasitoids failed to discriminate against hosts treated with LC_15_ or LC_35_  *Btk* in choice tests, but attacked fewer hosts treated with LC_70_  *Btk*, paralyzing and parasitizing more healthy hosts, and laying more eggs on them. In contrast, a no-choice test revealed that more hosts treated with LC_35_ and LC_70_  *Btk* were paralyzed compared with control or LC_15_-treated hosts, but the numbers of hosts parasitized and eggs laid did not vary among *Btk* treatments. Thus, females required an experience with healthy hosts, as they had in the choice test, to discriminate against diseased ones. We conclude that *H. hebetor* and *Btk* are compatible for joint application against *H. armigera*, which could potentially improve biological control of this pest.

The ectoparasitoid *Habrobracon hebetor* Say is an important biological control agent of *Helicoverpa armigera* Hubner and other lepidopteran pests (Ba et al. 2014, Mbata and Warsi 2019). This gregarious idiobiont ectoparasitoid has a fast growth rate, short generation time, high fecundity, and a broad host range, all of which contribute to its effectiveness as a biocontrol agent in integrated pest management (IPM) programs (Ghimire and Phillips 2014, Kabore et al. 2017). Typically, a female of *H. hebetor* first paralyzes several host larvae by stinging them and injecting them with venom. Once the venom has taken effect and paralyzed the larvae, the wasp returns to lay a variable number of eggs (ca. 8–30) on some of them, such that many more hosts are often paralyzed than are used for oviposition (Hagstrum and Smittle 1977, Antolin et al. 1995, Ghimire and Phillips 2014). Although this parasitoid is often released alone in augmentation biological control programs against lepidopterous pests of field crops in Iran, it can also be used in combination with entomopathogens such as *Bacillus thuringiensis* subsp*. kurstaki* Berliner (*Btk*) (Allahyari et al. 2020a).

The entomopathogen *Btk* has become an important biocontrol agent for *H. armigera* in many countries (Gujar 2005; Tabashnik et al. 2015, Nascimento et al. 2018). Because *H. hebetor* preferentially parasitizes late-instar caterpillars, whereas *Btk* is more effective against early instars (Sedaratian et al. 2014), the combined use of both agents is an attractive alternative to chemical insecticides for controlling *H. armigera*. However, the simultaneous application of a parasitoid and an entomopathogen raises questions about potential interactions between them, such as the ability of parasitoids to discriminate between healthy hosts and infected ones, and the consequences for progeny development when they do not. There are reports of *Btk* having negative impacts on the biology and life history of parasitoids (De Bortoli et al. 2017), including *Campoletis chlorideae* Uchida (Hymenoptera: Ichneumonidae) (Mohan et al. 2008), *Palmistichus elaeisis* Delvarre and LaSalle (Hymenoptera: Eulophidae) (Rolim et al. 2020), and also *H. hebetor* (Sedaratian et al. 2014).

*Bt* toxins have the potential to affect natural enemy development by diminishing the growth and vigor of their hosts, but can also make the host more vulnerable to attack by other natural enemies (Mohan et al. 2008). However, there exists variance among parasitoid species in susceptibility to *Bt* toxins; immature survival of *Meteorus pulchricornis* Wesmael (Hymenoptera: Braconidae) reared on larvae of *Spodoptera litura* F. (Lepidoptera: Noctuidae) fed diet amended with the field rate of *Btk* was not significantly different from controls (Walker et al. 2007). Similarly, Chilcutt and Tabashnik (1999) reported that *Btk* treatment of *Plutella xylostella* (Lepidoptera: Plutellidae) larvae had no effect on oviposition preference of the endoparasitoid *Cotesia plutellae* Kurdjumov (Hymenoptera: Braconidae), and direct feeding of the pathogen to wasps in honey had no detrimental effects. However, prolonged feeding of host larvae on *Btk*-contaminated diet was detrimental to parasitoid survival because of premature host mortality. Therefore, the ability of parasitoids to discriminate against infected hosts can be an important factor affecting parasitoid fitness, biocontrol efficacy, and persistence in the field when these agents are used in combination (Wan et al. 2019).

Parasitoid fitness is intrinsically linked to host quality because the latter affects juvenile survivorship, fecundity, and the body size of emerging adults (Vinson and Iwantsch 1980, Ghimire and Phillips 2014). Natural selection should favor parasitoids that maximize their fitness by distinguishing and avoiding unsuitable hosts (Jiang et al. 2014). The present study was conducted to quantify the negative effects of *Btk* on development and survival of *H. hebetor* when they parasitize larvae of *H. armigera* inoculated with various concentrations of *Btk* and to determine whether *H. hebetor* females are able to discriminate inoculated hosts from healthy ones. The results were expected to clarify the compatibility of these two biocontrol agents for joint application in integrated pest management programs for *H. armigera*.

## Materials and Methods

### Plant Culture

Three to five seeds of chickpea, *Cicer arietinum* L. (cv. Bivanij), were sown in each plastic pot (10 cm ht × 7 cm diam) filled with a mixture of soil (30%), coco peat (30%), peat moss (30%), and perlite (10%). The pots were held at a daytime temperature of 23 ± 3°C, lowered to 20 ± 3°C at night in a greenhouse at the Department of Horticulture, Ilam University. Plants were watered every 2–3 d, as required, and used in rearing and bioassays when they reached the six to eight leaf stages (8–12 d).

### Insect Colonies

Insect rearing and all experiments were carried out in climate-controlled chambers set to 28 ± 1°C, 60 ± 5% RH, and a 16:8 (L:D) photoperiod. An *H*. *armigera* colony was established by collecting larvae (ca. 250) from chickpea fields in Ilam Province, Iran, in the spring of 2018. Each larva was transferred to a ventilated plastic container (4 × 6 × 8 cm) and fed an artificial diet based on that of Twine (1971) with some modification: bean flour (205 g), yeast (35 g), wheat germ (30 g), sunflower oil (5 ml), formaldehyde 37% (2.5 ml), agar (14 g), ascorbic acid (3.5 g), methyl-para-hydroxyl-benzoate (2.2 g), sorbic acid (1.1 g), and distilled water (700 ml). Larvae were provided with a piece of diet (ca. 1 cm^3^) that was refreshed daily until they pupated. Emerged moths were transferred to a transparent Plexiglas oviposition chamber (20 × 30 × 30 cm), 30–40 per container, and provisioned daily with 10% honey solution provided on cotton balls in open Petri dishes (6 cm diameter) and allowed 4–5 d to mate. The top and walls of the chamber were covered with a fine mesh that served as an oviposition substrate. The mesh nets bearing eggs were collected daily and transferred to plastic bags held under the same physical conditions. After hatching, neonate larvae were transferred to potted chickpea plants (three or four per plant) and allowed to feed 4 d until they reached the second instar, whereupon they were either used in experiments or transferred to larval rearing containers (as above) to maintain the colony.

A laboratory culture of *H. hebetor* was maintained on late instars of *Ephestia kuehniella* (Zeller) obtained from the Plant Protection Bureau of Ilam Province. The parasitoid colony was established by collecting parasitized *H. armigera* larvae (ca. 60) or pupae (ca. 30) from chickpea fields in Ilam Province, Iran, in the spring of 2018. These were reared out under the same physical conditions as described above for *H. armigera*. Emerging wasps were transferred to plastic Petri dishes (9 cm diameter), fed with a 10% honey solution on strips of paper (1 × 3 cm). Generations were started by placing two male and two female wasps in each of a series of Petri dishes (as above) and providing each dish with 10–12 fifth-instar larvae of *E. kuehniella* daily. Adult wasps were introduced to clean Petri dishes with new hosts daily until both female wasps died. Parasitized host larvae were kept until emergence of adult wasps and the colony was reared for eight generations before use in the experiments. All experiments employed 3-d-old mated females that had each been provided with three late-instar larvae of *E. kuehniella* on each of two successive days to verify successful mating and oviposition prior to use.

### *Helicoverpa armigera* Bioassay

A wettable powder formulation of *Bacillus thuringiensis* subsp. *kurstaki* (Belthirul, 32,000 IU/mg) was obtained from Probelte Pharma, SL (Madrid, Spain). Five concentrations of *Btk* were prepared (250, 625, 1,250, 2,000, and 2,500 ppm), and in each treatment, the foliage of potted chickpea plants was immersed for 10 s in one of the *Btk* suspensions (*n* = 90 plants for each concentration), then air-dried on a laboratory bench for 10 min; control plants were immersed in distilled water. Each treated chickpea plant was then placed in a ventilated plastic container (12 cm diameter × 12 cm height). Second-instar *H. armigera* larvae were transferred to *Btk*-treated chickpea plants, one larva per plant. The basal container was covered with a second container (15 cm diameter × 13 cm height) ventilated by means of a fine mesh glued to an aperture cut in the bottom (7 cm diameter). Both containers had sloping sides, so that a larva-proof seal was obtained when the larger container was placed upside down over the smaller one (Allahyari et al. 2020b). The second-instar *H. armigera* larvae were then left to feed on the *Btk*-treated chickpea plants for 3 d under the same physical conditions as the insect colony. Larval mortality was recorded on day 3 post-inoculation, and lethal concentrations were determined using Probit analysis.

### Parasitoid Bioassay

After determining the lethal concentrations of *Btk* in the first bioassay, second-instar larvae of *H. armigera* were starved for ca. 8 h and then transferred to chickpea plants treated with one of three concentrations of *Btk* (LC_15_, LC_35_, and LC_70_), or distilled water as controls, one larva per plant, and allowed to feed for 72 h. In total, 85, 120, 250, and 60 larvae were fed plants treated with LC_15_, LC_35_, LC_70_, and water, respectively, in consideration of lower expected host survival at higher concentrations. Surviving larvae were isolated in plastic Petri dishes (6 cm diameter) and provisioned with artificial diet, as described above, for the remainder of their development. Experimental wasps were held as couples in Petri dishes for 2 d after emergence and provided *E. kuehniella* larvae to ensure successful mating. Next, two female wasps, each 3 d old, were transferred to each Petri dish containing a single *Btk*-treated or control larva of *H. armigera* for 24 h. After the wasps were removed, a single parasitoid egg was left on each larva and parasitized larvae were held under the standard experimental conditions until emergence of adult wasps. The duration of immature parasitoid development, numbers of pupae formed, and numbers of adults emerging were all recorded. After emergence, adults were transferred to Petri dishes (6 cm diameter) and provisioned with diluted honey (10%) on strips of paper (1 × 3 cm) every other day until death of the last wasp to estimate adult longevity.

### Choice Test

**Fig. 1.**
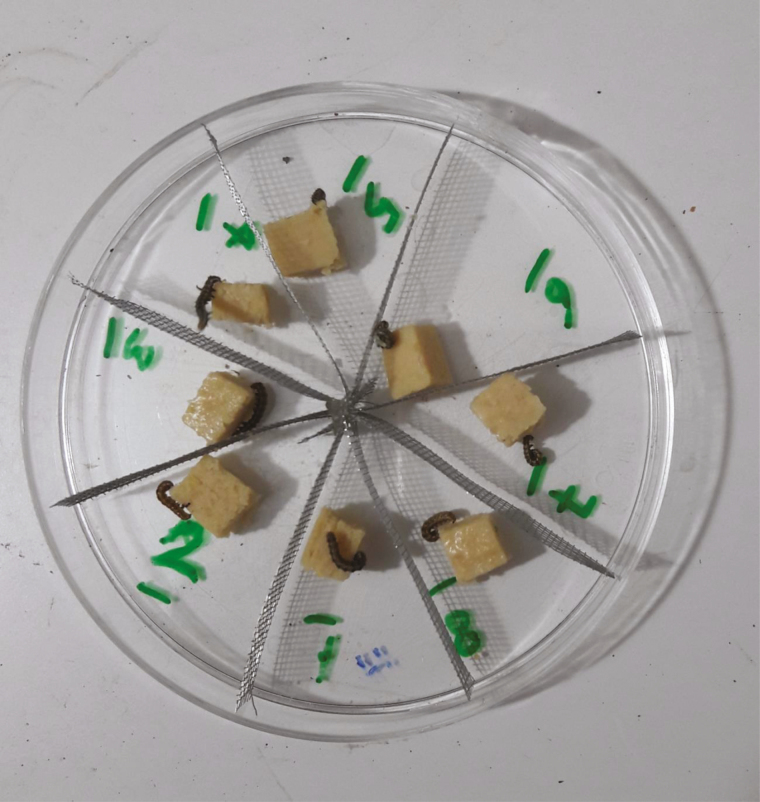
Choice test arena showing wire mesh dividers used to separate host larvae while allowing free passage of *Habrobracon hebetor* wasps between compartments.

Second-instar larvae of *H. armigera* were fed for 3 d on chickpea plants treated with one of three concentrations of *Btk* (LC_15_, LC_35_, and LC_70_), or distilled water as a control, as described above. Treated larvae that survived each of three *Btk* concentrations, and healthy larvae (controls), were isolated in plastic Petri dishes (9 cm diameter), which had been divided into eight equal radial sections by means of a wire mesh to isolate a single larva in each section and prevent cannibalism among them (Fig. 1). The wire mesh was coarse enough to permit wasps to move easily through the partitions. Four treated and four healthy larvae were arranged alternately among the eight sections of each dish, and each was provisioned with a piece of artificial diet (ca. 1 cm^3^). A single, 3-d-old female wasp was introduced to each Petri dish and allowed forage for 24 h, whereupon the wasp was removed and all paralyzed and parasitized hosts, as well total eggs laid, were recorded. If a larvae was still alive but showed no movement when stimulated by a fine brush, it was considered to be paralyzed and parasitized if it received at least one egg (all parasitized larvae were paralyzed, but not all paralyzed larvae were parasitized). Altogether, 60 female wasps were tested at each concentration, one female per replicate. Data for replicates in which the wasp was killed by the host larvae were discarded.

### No-Choice Test

A no-choice test was conducted using the same procedures as the choice test, except that eight *Btk*-treated larvae from each of three concentrations (LC_15_, LC_35_, and LC_70_) or eight healthy larvae (as control) were placed in each Petri dish (9 cm diameter) and a 3-d-old female wasp was introduced to each (*n* = 60) as described above. Data for replicates in which the wasp were killed by the host larvae during the experiment were discarded.

### Statistical Analyses

Lethal concentrations of *Btk* were calculated by SPSS software package (SPSS 1998) using mortality data of the bioassay subjected to Probit analysis (Finney 1971). Parasitoid survival was analyzed by Kaplan–Meier (α = 0.05). Developmental times were compared by one-way analysis of variance (ANOVA; SPSS 1998), and Fisher’s least significant difference (LSD) test was used to separate means (α = 0.05). Mean numbers of paralyzed and parasitized larvae and numbers of eggs laid in the choice test were compared by paired *t*-test (two tailed). In the nonchoice test, the data were analyzed by one-way ANOVA (SPSS 1998) followed by Fisher’s LSD test to separate means (α = 0.05).

## Results

### *Helicoverpa armigera* Bioassay

The lethality of *Btk* against second-instar larvae of *H. armigera* was measured after 72 h of continuous feeding on treated chickpea plants and increased significantly with the concentration applied (Table 1).

**Table 1.** Lethality of various concentrations of *Bacillus thuringiensis* var. *kurstaki* against second-instar larvae of *Helicoverpa armigera* (*n* = 90 per concentration) derived from Probit analysis (df = 3, intercept = 6.7 ± 0.62) with 95% confidence intervals (CIs)

Lethality	Concentration (ppm)	95% CIs	χ ^2^	*P*
LC_15_	201.9	147.8–254.7	4.96	0.174
LC_35_	371.7	301.3–438.7		
LC_70_	871.7	762.4–1,003.0		

### Parasitoid Bioassay

**Fig. 2.**
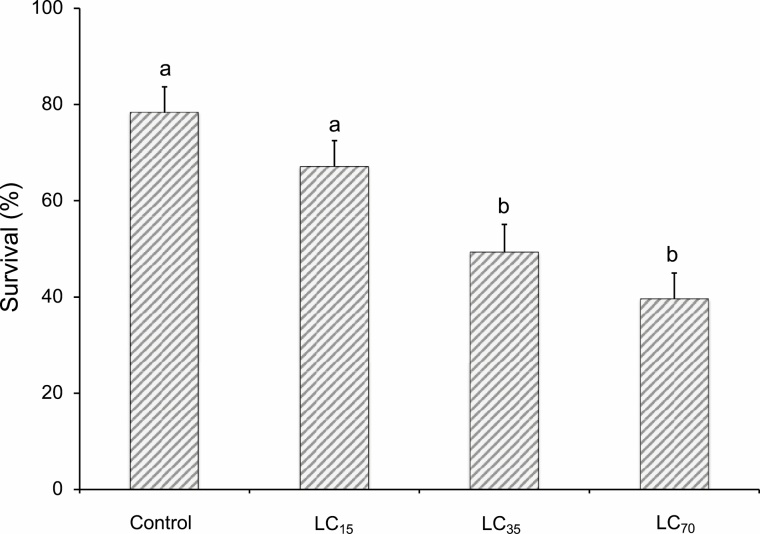
Emergence success (mean + SE survival, in percent) of solitary *Habrobracon hebetor* larvae that developed on *Helicoverpa armiger*a larvae that survived 72-h feeding on *Btk*-treated chickpea plants as second instars. Columns bearing the same letter were not significantly different (ANOVA followed by Fisher’s LSD, α = 0.05).

When second-instar *H. armigera* larvae fed for 72 h on chickpea plants treated with different concentrations of *Btk*, survival was 100% (60/60) in controls, compared with 89.4% (76/85) in the LC_15_ treatment, 62.5% (75/120) in the LC_35_ treatment, and 19.2% (48/250) in the LC_70_ treatment. When surviving *H. armigera* larvae were each parasitized with a single egg, parasitoid survival was significantly reduced in the LC_70_ and LC_35_ treatments, whereas the LC_15_ treatment survival was not significantly different from controls (Fig. 2). Although the incubation period of parasitoid eggs hatching on *H. armigera* larvae was not affected by *Btk* treatment (*F*_3,255_ = 0.18, df = 3,255; *P* = 0.910), larval development was significantly delayed in the LC_70_ treatment (*F* = 6.54, df = 3,155; *P* < 0.001) and total immature development was delayed by both the LC_70_ and LC_35_ treatments, but not by the LC_15_ treatment (*F* = 5.12, df = 3,153; *P* = 0.002; Table 2). Pupation time was not significantly different among treatments (*F* = 1.63, df = 3,153; *P* = 0.190), but the longevity of male (*F* = 3.03, df = 3,83; *P* = 0.030) and female (*F* = 2.78, df = 3,69; *P* = 0.048) wasps was significantly reduced in the LC_70_ and LC_35_ treatments.

**Table 2.** Mean (± SE) duration of immature stages and adult longevities (in days) of *Habrobracon hebetor* when reared on *Helicoverpa armigera* larvae which fed on *Btk*-treated chickpea plants for 72 h

		Treatment						
Life stage	*n*	Control	*n*	LC_15_	*n*	LC_35_	*n*	LC_70_
Egg	60	1.3 ± 0.06 a	76	1.3 ± 0.05 a	75	1.3 ± 0.05 a	48	1.3 ± 0.07 a
Larva	47	2.3 ± 0.09 c	51	2.5 ± 0.10 bc	37	2.7 ± 0.14 b	19	3.1 ± 0.15 a
Pupa	47	5.7 ± 0.10 a	51	5.9 ± 0.13 a	37	6.1 ± 0.14 a	19	6.1 ± 0.21 a
Total immature	47	9.3 ± 0.13 c	51	9.7 ± 0.20 bc	37	10.1 ± 0.24 ab	19	10.5 ± 0.28 a
Male longevity	26	4.4 ± 0.37 a	28	3.6 ± 0.35 ab	19	3.1 ± 0.42 b	11	2.7 ± 0.52 b
Female longevity	21	10.7 ± 0.72 a	23	9.0 ± 0.94 ab	18	7.3 ± 1.04 b	8	6.9 ± 1.54 b

Means within rows followed by the same letter were not significantly different among treatments (ANOVA followed by Fisher’s LSD, α = 0.05).

### Choice Test

Female parasitoids failed to discriminate between healthy hosts and those treated with the LC_15_ of *Btk*, paralyzing and parasitizing similar numbers of them, and laying similar numbers of eggs on them (Table 3). When LC_35_-treated hosts were available, females paralyzed more treated hosts than healthy ones, but parasitized equal numbers of them and laid similar numbers of eggs on them. When LC_70_-treated hosts were available, parasitoids paralyzed and parasitized more healthy hosts than treated ones and laid more eggs on them.

**Table 3.** Mean (± SE) numbers of *Helicoverpa armigera* larvae (out of *n* = 8 per female) paralyzed and parasitized by *Habrobracon hebetor* (*n* = 60 per treatment) in a 24-h choice test, and total number of eggs laid

	Host type			
Variable	Healthy	Treated	*t*	*P*
LC_15_ (df = 53)				
No. of paralyzed	2.0 ± 0.14	2.0 ± 0.10	0.32	0.751
No. of parasitized	1.4 ± 0.10	1.3 ± 0.09	0.73	0.471
No. of eggs laid	10.4 ± 0.77	9.2 ± 0.67	1.29	0.204
LC_35_ (df = 56)				
No. of paralyzed	1.9 ± 0.11	2.3 ± 0.09	2.42	0.019
No. of parasitized	1.1 ± 0.07	1.3 ± 0.07	1.22	0.226
No. of eggs laid	11.0 ± 0.70	11.2 ± 0.66	0.23	0.819
LC_70_ (df = 57)				
No. of paralyzed	2.2 ± 0.11	1.9 ± 0.11	2.00	0.050
No. of parasitized	1.6 ± 0.68	1.0 ± 0.94	4.33	<0.001
No. of eggs laid	12.7 ± 0.88	9.1 ± 0.75	3.34	0.001

Treated host larvae were second instars that fed for 72 h on chickpea plants treated with *Btk*; healthy larvae were fed on plants treated with distilled water (paired *t*-test, two tailed).

### No-Choice Test

In a no-choice situation, similar numbers of hosts were paralyzed in the LC_15_ treatment as in controls, but more hosts were paralyzed in the LC_35_ and LC_70_ treatments (Table 4). However, paralyzed hosts in LC_35_ were not significantly different from those in LC_70_. There were no differences among treatments in the numbers of hosts parasitized or the numbers of eggs laid.

**Table 4.** Mean (± SE) numbers of *Helicoverpa armigera* larvae (out of *n* = 8 per female) paralyzed and parasitized by *Habrobracon hebetor* females (*n* = 60 per treatment) and numbers of total eggs laid on the healthy (control) or treated hosts in a no-choice test

Host treatment	No. of hosts paralyzed	No. of hosts parasitized	No. of eggs laid
Control	3.8 ± 0.21 b	2.8 ± 0.16 a	19.9 ± 1.4 a
LC_15_	3.7 ± 0.17 b	2.6 ± 0.14 a	21.1 ± 1.4 a
LC_35_	4.4 ± 0.15 a	2.9 ± 0.14 a	20.8 ± 1.2 a
LC_70_	4.3 ± 0.13 a	2.6 ± 0.13 a	20.3 ± 1.2 a
*F*	7.02	1.07	0.16
df	3,230	3,230	3,230
*P*	0.005	0.363	0.925

Host larvae were fed on *Btk*-treated chickpea plants for 72 h, beginning as second instars, with control plants treated with distilled water. Means bearing different letters are significantly different within columns (one-way ANOVA followed by Fisher’s LSD, α = 0.05).

## Discussion

Female parasitoids did not discriminate against second-instar *H. armigera* treated with the LC_15_ concentration of *Btk* in either choice or no-choice tests, and similar numbers were paralyzed and parasitized as healthy ones. This would suggest that parasitoids did not detect any symptoms of *Btk* infection in these hosts, which is ostensibly characterized by sluggish movement and weight loss (Blumberg et al. 1997). However, hosts treated with LC_35_  *Btk* were paralyzed more often than healthy hosts in both choice and no-choice bioassays, suggesting that their infections rendered them more vulnerable to initial attacks by the parasitoid, without rendering their behavior sufficiently abnormal to cue wasp avoidance, even when they were presented with healthy alternatives. Host defensive responses represent a hazard to wasps, as these large host larvae are sometimes able to injure or kill attacking parasitoids (Mohan et al. 2008). Hosts treated with LC_70_ were also paralyzed more often than controls in the no-choice test, but the reverse was true in the choice test, suggesting that disease symptoms in this treatment were pronounced enough that wasps were more likely to avoid them when given a chance to compare them to healthy hosts. Allahyari et al. (2020a) showed that *H. armigera* larvae inoculated with sublethal concentrations of a nucleopolyhedrovirus, HearNPV, were more often paralyzed by *H. hebetor* females than controls and inferred reduced defensive responses in infected hosts. Sometimes, *Bt*-infected hosts can be more susceptible to parasitism than untreated hosts, due to their slower development and smaller size (Mascarenhas and Luttrell 1997, Erb et al. 2001). However, *H. hebetor* females in the no-choice test parasitized similar numbers of LC_35_-treated and LC_70_-treated hosts as they did healthy ones, and laid equal numbers of eggs on each. Therefore, whatever infection-related factors increased their susceptibility to attack did not alter their acceptability for oviposition for these females.

Parasitoid females discriminated against hosts treated with the LC_70_  *Btk* concentration in the choice test and paralyzed and parasitized fewer of them compared with healthy hosts. We conclude that these hosts inoculated with higher doses of *Btk* displayed sufficiently abnormal symptoms that their acceptability to parasitoids for oviposition was reduced. However, they were parasitized at similar rates and received similar numbers of eggs in the no-choice test. The different results of these two tests suggest that females require experiences with healthy hosts, as they had in the choice test, to discriminate disease symptoms in infected ones. Similarly, Akinkurolere et al. (2009) reported that female *H. hebetor* parasitized more less-preferred, early-instar larvae of *Plodia interpunctella* Hubner (Lepidoptera: Pyralidae) when they were offered in no-choice situations, having had no experience with larger ones, and Wan et al. (2019) found that *Microplitis pallidipes* Szepligeti (Hymenoptera: Braconidae) required prior experience with healthy larvae of *Spodoptera exigua* (Lepidoptera: Noctuidae) before they could discriminate against those with nucleopolyhedrosis virus (NPV) infections.

Development and survival of *H. hebetor* larvae were both negatively affected when they developed on hosts subjected to the LC_70_ and LC_35_  *Btk* treatments. Although immature development was delayed by less than 1 d, the adult longevity of wasps was also reduced significantly. It has been argued that natural selection should favor the avoidance of entomopathogen-infected hosts, which should select for parasitoids that can distinguish them from healthy ones (Flexner et al. 1986, Jiang et al. 2014). For example, both the growth and immature survival of the solitary endoparasitoid *C. chlorideae* were reduced in a dosage-dependent manner, relative to controls, when their *H. armigera* larval hosts fed continuously on a diet contaminated with sublethal concentrations of *Btk* (Mohan et al. 2008). Similarly, Rolim et al. (2020) reported negative impacts on *P. elaeisis* when this endoparasitoid developed in host larvae of *Spodoptera frugiperda* (Smith) (Lepidoptera: Noctuidae), many of which extended to the subsequent generation. These impacts included impaired foraging behavior and reduced reproduction and longevity that the authors inferred would reduce compatibility between these biocontrol agents.

These various studies suggest that infection with *Bt* will reduce the suitability of lepidopteran hosts for parasitism, but provided the hosts survive long enough for completion of wasp development, the parasitoid can survive. Sedaratian et al. (2014) examined *H. hebetor* larvae developing on *H. armigera* larvae treated with sublethal concentrations of *Btk* and found negative effects on their development, survival, and reproductive rate, which was reflected in diminished life table parameters such as finite and intrinsic rates of increase. However, they did not assess wasp behavioral responses to inoculated hosts. *Bt*-infected hosts may be smaller and provide poorer nutrition for parasitoid growth and development (Romeis et al. 2006), so a failure to discriminate against infected hosts under field conditions could potentially impair parasitoid reproductive success and reduce fitness. Our behavioral observations indicate that *H. hebetor* has some ability to discriminate against infected hosts, but it depends on both the degree of infection and whether the wasp has an opportunity to compare them to healthy hosts. Given that foliar applications of *Btk* have relatively short persistence in the field, many pest larvae will probably consume sublethal doses and remain as suitable hosts for parasitism by *H. hebetor*. Any ability of wasps to discriminate against heavily infected hosts could aid biological control outcomes by shifting more parasitism toward uninfected and sublethally infected host larvae.

Furthermore, laboratory studies have shown that *H. armigera* has the ability to evolve resistance to Btk (Lu et al. 2004), and resistant populations have been detected in the field (Liu et al. 2010). This eventuality might be delayed or precluded if releases of *H. hebetor* reduce the survival of resistant genotypes. Allahyari et al. (2020b) found evidence of additivity between *Btk* and *H. hebetor* in controling *H. armigera* on chickpea in an earlier field study, which would support their effectiveness in joint application. Thus, even though *Btk* had some negative effects on *H. hebetor* under laboratory conditions, and the parasitoid failed to discriminate against hosts treated with low concentrations of *Btk*, we conclude that these two biocontrol agents are potentially compatible for combined application against *H. armigera* under field conditions.
